# The influence of sex steroids on cognition in elderly men

**DOI:** 10.1016/j.clinsp.2026.100892

**Published:** 2026-02-28

**Authors:** Carolina C.B. Caetano, Sami Liberman, Jéssica M.L. Ie, Valmari C.A. Toscano, Wilson Jacob, Tânia A.S.S. Bachega

**Affiliations:** a Disciplina de Geriatria, Hospital das Clínicas, Faculdade de Medicina, Universidade de São Paulo, São Paulo, SP, Brazil; b Unidade de Adrenal, Laboratório de Hormônios e Genética Molecular LIM 42, Disciplina de Endocrinologia e Metabologia, Hospital das Clínicas, Faculdade de Medicina, Universidade de São Paulo, São Paulo, SP, Brazil

**Keywords:** Cognition, Elderly, Testosterone levels, Estradiol levels, GDS, MMSE

## Abstract

**Background:**

Testosterone plays a crucial role in cognitive development and behavioral differences between sexes. However, it remains unclear whether age-related testosterone decline affects cognitive function in elderly men.

**Objective:**

To determine whether serum steroid levels influence cognitive function in elderly males.

**Methods:**

Sixty men with a mean age of 73.5 ± 5.9 years were evaluated. Global cognitive function was assessed using the Mini-Mental State Examination (MMSE), administered by a single trained examiner. To rule out the influence of depression on MMSE, the Geriatric Depression Scale (GDS) was applied. Age, BMI, education level, serum Total Testosterone (TT), Free Testosterone (FT), Estradiol (E2), and sex hormone-binding globulin levels were measured and correlated with total and subdomain MMSE scores and GDS scores. Nonparametric tests and multivariable regression analyses were performed.

**Results:**

Age was negatively correlated with TT (*r* = -0.36; *p* = 0.003), and TT was inversely associated with BMI (*r* = -0.45; *p* < 0.001). BMI was negatively correlated with total MMSE scores (*r* = -0.30; *p* = 0.010). TT and E2 levels showed no significant associations with overall MMSE, except for a positive correlation between TT and the Spatial Orientation subdomain (*r* = 0.26; *p* = 0.040). TT and FT were both negatively correlated with GDS scores (*r* = -0.32; *p* = 0.010; and *r* = -0.27; *p* = 0.030, respectively). After adjustment, only education years remained independently associated with MMSE (β = 0.374, *p* = 0.013).

**Conclusions:**

Although lower serum testosterone levels were associated with depressive symptoms, no significant correlation was found with global cognitive performance; an interesting finding was the impact of years of education on MMSE score.

## Highlights

• Education years predicted global cognitive performance in older men.
• Serum testosterone levels inversely correlated with depression scores.
• Higher BMI was associated with lower MMSE scores in bivariate analysis.
• BMI was negatively correlated with serum testosterone levels.

## Introduction

The growing elderly population is a global phenomenon that was once primarily associated with developed countries, but nowadays, it is occurring in nearly every nation. Current data from the Brazilian Institute of Geography and Statistics show that life expectancy has increased more than four years over the 2010 indicator, to approximately 74.38-years among males^1^^,^^2^ and by 2050, individuals over 60-years old are projected to make up one-fifth of the global population.^3^

Aging is associated with increased prevalence of chronic diseases, including cognitive function deficits, and the number of people affected by dementia is projected to reach 131-million in the next 25-years. Cognitive function includes multiple domains, such as memory, language, calculation and spatial ability, which can be measured through a variety of standardized tests. Among these domains, memory is the most impaired with aging.^3^

Deficits in cognition and depression are among the main mental health problems in the elderly. Meta-analyses indicate that the prevalence rates of depressive symptomatology are 17.1 % in individuals aged 75-years and older, and 19.5 % in individuals aged 50 and.^3^ Early detection of depressive disorders in older adults is essential to prevent increased morbidity and reduced quality of life. Persistent depressive symptoms lead to cognitive impairments and may also increase the risk of both suicidal and non-suicidal mortality, including deaths related to cardiac conditions.^4^

In the literature, it is discussed whether sex hormones may influence cognition in the elderly, but their relationship with cognition is complex.^5^ Testosterone receptors are found in the medial preoptic area, ventromedial hypothalamus, medial amygdala, nucleus accumbent, stria terminalis end septum, and cerebral cortex. Both testosterone and estrogen are related to memory, executive function and play a neuroprotective function.^6^^,^^7^ Additionally, testosterone is aromatized into estrogen, which acts via estrogen receptors, suggesting that testosterone may influence brain function either directly or through the estrogenic pathway.^8^

During aging, total testosterone levels typically undergo a decline during the third or fourth decade of life, decreasing at a rate of 0.2 % to 1 % annually. Subcomponents of testosterone, such as free testosterone, experience even more rapid declines, ranging from 2 % to 3 % annually.^9^ It is unknown whether this decline is responsible for alterations observed during the physiological aging process. To support this hypothesis, individuals with Late-Onset Hypogonadism (LOH) exhibit changes similar to those seen in senescence, such as reduced sexual function and desire, fatigue, poor concentration, and depression. They also present with obesity, decreased muscle mass and bone mineral density, as well as alterations in the hematopoietic system.^10^

Interestingly, recent studies suggest that the reduction in androgen levels, such as testosterone, may be a consequence of cognitive decline rather than its cause, the physiopathology is unknown. This hypothesis arises from observations that, while both cognitive function and androgen levels decline with age, life-long hypogonadism is not typically associated with cognitive decline; besides, cognitive impairment does not consistently improve with testosterone replacement therapy.^11^, ^12^, ^13^

There is no data from the Brazilian population regarding the association of serum steroid levels during aging and cognitive subdomain functions. Therefore, the aim of the present multidisciplinary study was to evaluate the Mini-Mental State Examination (MMSE) scores in a cohort of elderly men, followed in a single center, and to determine whether serum testosterone levels influence their performance in these tests.

## Materials and methods

### *Study design and participants*

This cross-sectional observational study included 60 clinically stable male participants aged 60-years or older, recruited from the Geriatrics Service of the Hospital das Clínicas, Faculdade de Medicina da Universidade de São Paulo. All individuals signed a written informed consent form prior to participation. The study was approved by the Ethics Committee for Research Project Analysis – CAPPesq (approval #864.909) and by the Sectorial Commission of Psychology Ethics. This study followed the Strengthening the Reporting of Observational Studies in Epidemiology (STROBE) guidelines.

Inclusion criteria were male sex, age ≥ 60-years, and preserved global functionality. Participants were excluded if they had reduced mobility, communication impairments, dementia, major depression, psychosis, or any condition involving delusions or hallucinations, based on DSM-5 diagnostic criteria.^14^ Individuals with uncontrolled diabetes mellitus, hypothyroidism, or heart failure were also excluded, as were those using central nervous system medications, antidepressants, or sex steroid replacement. Active smokers were not included.

Clinical and lifestyle data were collected through structured interviews. The level of physical activity was assessed using the International Physical Activity Questionnaire (IPAQ), validated in Brazil,^15^ revealing that 72.8 % of participants were considered sedentary. Despite advanced age, 72.8 % were still engaged in some form of professional activity. Regarding morbidity burden, 57.6 % of the sample had more than two chronic diseases. The most common conditions were systemic arterial hypertension (55.0 %), diabetes mellitus (33.3 %), cardiomyopathy (28.3 %), dyslipidemia (45.0 %), and hypothyroidism (15.0 %).

Polypharmacy was frequent, with 84.5 % of participants reporting the use of five or more medications. The most common medications included angiotensin II receptor blockers, angiotensin-converting enzyme inhibitors, diuretics, metformin, sulfonylureas, statins, and levothyroxine. All chronic diseases were considered clinically and metabolically controlled at the time of assessment; mean fasting blood glucose levels were 107±24.4 mg/dL (range: 78–176 mg/dL), glycated Hemoglobin (HbA1c) 5.8 ± 0.8 % (range: 4.5–7.8), and TSH 2.7 ± 0.9 µIU/mL (range: 1.0–3.9).

### *Assessments of cognition and depression*

Cognitive function was evaluated using the Mini-Mental State Examination,^16^ a 30-item instrument that provides information in the following areas: Temporal Orientation (TO), Spatial Orientation (SO), Immediate Memory (IM), Attention and Calculation (AC), Delayed Recall (DR), Language (Lang), and Constructive Visual Ability (CVA). MMSE scores range from 0 to 30 points and are strongly influenced by education; therefore, population-adjusted cutoffs were applied.^17^ For this study, MMSE scores were also analyzed by subdomains.

Depressive symptoms were assessed using the 15-item Geriatric Depression Scale (GDS-15), which is a reliable and widely validated tool for mood screening in older adults. A cutoff score of ≥ 5 was used to indicate relevant depressive symptoms, based on values adapted to the Brazilian population.^18^^,^^19^ Both instruments were administered by a single trained psychologist blinded to hormonal data.

### *Hormone assays*

Serum total testosterone and estradiol levels were measured by commercial radioimmunoassay kits. LH and FSH levels were measured by immunofluorimetric assay kits. SHBG levels were measured by an immunoradiometric assay kit. Free testosterone levels were based on total serum testosterone and SHBG levels. All interassay and intrassay coefficients of variation were less 11 %.

### *Statistical analysis*

Data was analyzed using SPSS (version 27, IBM Corp., Armonk, NY) with a significance level set at *p* < 0.05 and 95 % Confidence Intervals (95 % CI). Data distribution was assessed using the Kolmogorov-Smirnov test. Given the non-parametric nature of several variables, descriptive results were expressed as median (interquartile range) or mean ± standard deviation, according to distribution.

The Spearman correlation test was used to explore associations between serum sex steroid levels (total testosterone, free testosterone, estradiol, and sex hormone-binding globulin) and cognitive performance (MMSE total and its subdomains), depressive symptoms (GDS), and anthropometric variables (BMI).

To compare cognitive and depressive outcomes across testosterone level strata (≤ 25th percentile, 25th–75th percentile, ≥ 75th percentile), the Kruskal-Wallis test was applied. However, testosterone levels were also treated as continuous variables in multivariate regression analyses to preserve statistical power and avoid arbitrary categorization.

Multivariable linear regression models were constructed to identify independent associations between hormonal variables and outcomes (MMSE and GDS). Covariates included age, education, BMI, and chronic diseases such as systemic arterial hypertension, diabetes mellitus, and cardiac insufficiency. Variables with *p* < 0.20 in univariate analyses were entered into the models. The GDS outcome was also explored using Generalized Linear Models (GLM) with Poisson distribution and identity link function, as GDS scores followed a discrete distribution with positive skewness.

To the risk of false-positive findings due to multiple comparisons ‒ particularly in MMSE subdomain analyses ‒ a Bonferroni correction was applied where appropriate (adjusted p-values). All assumptions for linearity, homoscedasticity, multicollinearity, and residual distribution were tested and met. The data supporting the findings of this study can be requested from the corresponding author.

## Results

The study included 60 elderly men with a mean age of 73.5-years (SD ±5.9) and an average educational level of 9.5-years (SD ±4.5). The mean BMI was 26.9 kg/m^2^ (SD ±3.9), with 32 % of participants classified as having normal weight (BMI ≤ 25), 55 % as overweight (BMI > 25 and < 30), and 13 % as obese (BMI ≥ 30). The most frequent comorbidities were systemic arterial hypertension (55 %), dyslipidemia (45 %), and diabetes mellitus (33 %), followed by heart failure (22 %) and hypothyroidism (15 %). Regarding hormonal profiles, the mean total testosterone level was 431.6 ng/dL (SD ±179.6), free testosterone averaged 7.58 pmoL/L (SD ±2.8), and estradiol levels were 27.1 pg/mL (SD ±9.7). Overall, the sample presented clinical and metabolic characteristics consistent with an older male population under stable outpatient care. Baseline clinical, anthropometric, and hormonal characteristics are summarized in Table 1.

**Table 1:** Clinical, anthropometric, and hormonal characteristics of elderly men included in the study.

	Mean ± SD or n ( %)
**Age (years)**	73.5 ± 5.9
**Education (years)**	9.5 ± 4.5
**BMI (kg/m^2^)**	26.9 ± 3.9
BMI ≤ 25	19 (32 %)
BMI > 25 and < 30	33 (55 %)
BMI ≥ 30	8 (13 %)
**Systemic arterial hypertension**	33 (55 %)
**Diabetes mellitus**	20 (33 %)
**Heart failure**	13 (22 %)
**Dyslipidemia**	26 (45 %)
**Hypothyroidism**	8 (15 %)
**Total testosterone (ng/dL)**	431.6 ± 179.6
**Free testosterone (pmoL/L)**	7.58±2.8
**Estradiol (pg/mL)**	27.1 ± 9.7

**Fig. 1:**
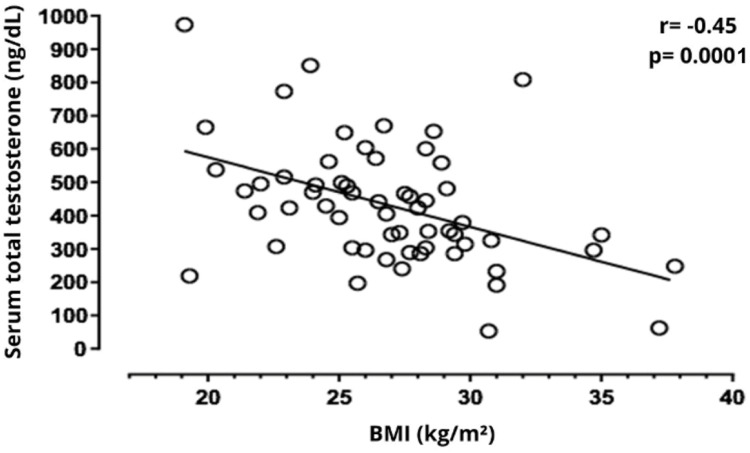
Serum testosterone total levels presented inversal correlation with BMI.

**Fig. 2:**
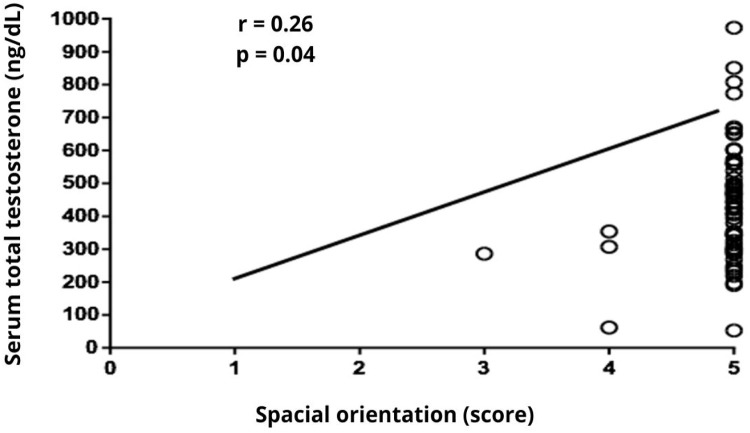
Serum testosterone total levels showed an positive correlation with spatial orientation scores.

Significant correlations were observed among clinical variables, cognitive domains, and serum steroid levels in this sample of elderly men. Age was negatively correlated with serum total testosterone (*r* = −0.36, *p* = 0.003), and free testosterone levels (*r* = −0.32, *p* = 0.010), while positively correlated with GDS scores (*r*= 0.30, *p* = 0.016). Education showed a strong positive correlation with serum total testosterone (*r* = 0.48, *p* < 0.001) and estradiol levels (*r* = 0.26, *p* = 0.040), and a negative correlation with BMI (*r*= −0.40, *p* = 0.001). BMI was inversely correlated with MMSE total score (*r*= −0.30, *p* = 0.010), language (*r* = −0.25, *p* = 0.040), and total testosterone (*r* = −0.45, *p* < 0.001, Fig. 1). Total testosterone levels were positively correlated with free testosterone (*r* = 0.68, *p* < 0.001) and spatial orientation (*r* = 0.26, *p* = 0.040, Fig. 2) and negatively correlated with GDS scores (*r* = −0.32, *p* = 0.010). Free testosterone was also positively associated with estradiol (*r* = 0.38, *p* = 0.002) and negatively with GDS scores (*r* = −0.27, *p* = 0.030). These findings suggest a multifaceted relationship between endocrine status, mood, and cognitive domains ‒ particularly spatial orientation and language ‒ with education and BMI emerging as important covariates (Table 2).

**Table 2:** Correlations among clinical variables, cognitive function and serum steroid levels in elderly men.

		AGE	EDU	BMI	MMSE	TO (0‒5)	SO (0‒5)	IM (0‒3)	AC (0‒5)	DR (0‒3)	LANG (0‒8)	CVA (0‒1)	GDS	E2	TT	FT
**Spearman *r***	**Age**		**−0,40**	0.12	−0.19	−0.11	−0.20	0.01	−0.17	−0.07	−0.22	−0.11	**0.30**	**−0.24**	**−0.36**	**−0.32**
**p-value (two-tailed)**			**0.001**	0.350	0.140	0.370	0.12	0.930	0.180	0.540	0.080	0.370	**0.016**	**0.060**	**0.003**	**0.010**
**Spearman r**	**TT**	**−0.36**	**0.48**	**−0.45**	0.17	−0.04	**0.26**	0.05	0.09	0.19	0.15	0.04	**−0.32**	**0.38**		**0.68**
**p-value (two-tailed)**		**0.003**	**<0.001**	**<0.001**	0.170	0.730	**0.040**	0.690	0.470	0.120	0.230	0.710	**0.010**	**0.002**		**<0.001**
**Spearman *r***	**FT**	**−0.32**	0.22	−0.14	−0.03	−0.18	0.21	−0.07	−0.02	0.13	−0.12	0.08	**−0.27**	0.38	**0.68**	
**p-value (two-tailed)**		**0.010**	0.080	0.270	0.800	0.150	0.100	0.550	0.820	0.310	0.360	0.540	**0.030**	**0.002**	**<0.001**	
**Spearman *r***	**E2**	**−0.24**	**0.26**	0.02	0.14	−0.02	0.11	0.23	0.00	0.17	0.09	0.20	−0.23		**0.37**	**0.38**
**p-value (two-tailed)**		**0.060**	**0.040**	0.850	0.270	0.870	0.400	0.070	0.950	0.180	0.460	0.110	0.070		**0.003**	**0.002**
**Spearman *r***	**BMI**	0.12	**−0.40**		**−0.30**	−0.24	−0.11	−0.04	−0.18	−0.22	**−0.25**	0.02	0.15	−0.04	**−0.45**	−0.14
**p-value (two-tailed)**		0.350	**0.001**		**0.010**	0.060	0.360	0.720	0.150	0.080	**0.040**	0.870	0.240	0.760	**<0.001**	0.270

TT, Total Testosterone; FT, Free Testosterone; BMI, Body Mass Index; SHBG, Sex Hormone-Binding Globulin; EDU, Education; MMSE, Mini-Mental State Examination; TO, Temporal Orientation; SO, Spatial Orientation; IM, Immediate Memory; AC, Attention and Calculation; DR, Delayed Recall; LANG, Language; CVA, Constructive Visual Ability; GDS, Geriatric Depression Scale; E2, Estradiol.

Table 3 shows the distribution of cognitive performance (MMSE and subdomains), depressive symptoms (GDS), and education level across tertiles of total testosterone among 60 elderly men. A statistically significant difference was observed for years of education (*p* = 0.001), with Bonferroni-corrected pairwise comparisons indicating significance between G1 and G3 (*p* = 0.001). MMSE total scores and individual cognitive domains did not differ significantly among the groups. GDS scores showed an overall difference (*p* = 0.040), but post-hoc tests did not reveal statistically significant differences between pairs after Bonferroni correction.

**Table 3:** MMSE and GDS scores according to serum total testosterone levels.

	G1 ≤ 25th (303 ng/dL)	G2 25th/75th (304‒516)	G3 ≥ 75th (517 ng/dL)	p
	TT	TT	TT	
**n**	16	29	15	
**Education yrs.**	6.6 ± 3.9	10.3 ± 4.3	11.5 ± 3.8	**0.001**
**MMSE**	26.5 ± 3.1	26.9 ± 2.5	27 ± 3.4	0.740
**TO**	4.7 ± 0.5	4.9 ± 0.3	4.7 ± 0.4	0.090
**SO**	4.8 ± 0.5	4.9 ± 0.2	5 ± 0	0.370
**IM**	2.8 ± 0.5	2.9 ± 0.3	3 ± 0	0.620
**AC**	4.0 ± 1.5	3.2 ± 1.9	3.9 ± 1.8	0.250
**DR**	2.0 ± 0.9	2.4 ± 0.7	2.4 ± 1.0	0.240
**LANG**	7.6 ± 0.5	7.8 ± 0.3	7.5 ± 1.0	0.190
**CVA**	26.5 ± 3.1	0.5 ± 0.5	0.4 ± 0.5	0.440
**GDS**	3.4 ± 3.1	2.3 ± 2.2	1.3 ± 0.5	**0.040**

TT, Total Testosterone; MMSE, Mini-Mental State Examination; TO, Temporal Orientation; SO, Spatial Orientation; IM, Immediate Memory; AC, Attention and Calculation; DR, Delayed Recall; LANG, Language; CVA, Constructive Visual Ability; GDS, Geriatric Depression Scale. Education yrs. Bonferroni-corrected p-value: G1 vs. G2 = 0.152; G1 vs. G3 = 0.001; G2 vs. G3 = 0.070; GDS Bonferroni-corrected p-value: G1 vs. G2 = 0.999; G1 vs. G3 = 0.758; G2 vs. G3 = 0.999.

The authors examined the association between global cognitive performance (MMSE scores) and clinical as well as hormonal variables using both unadjusted and adjusted linear regression models. The adjusted model included age, education, BMI, estradiol, total testosterone, free testosterone, and SHBG levels. In the unadjusted analysis, years of education were significantly associated with MMSE scores (β = 0.374, *p* = 0.013). After adjustment, education remained independently associated with MMSE (β = 0.398, *p* = 0.029), while no significant associations were observed for age, BMI, or hormonal parameters. These results reinforce the role of educational background as a key determinant of cognitive performance in older adults (Table 4).

**Table 4:** Unadjusted and adjusted analyses for the influence of hormonal and clinical variables on MMSE scores in 60 elderly men.

Variable	Unadjusted		Adjusted	
	β (SE)	p	β (SE)	p
Age (years)	−0.133 (0.117)	0.255	−0.041 (0.149)	0.783
Education (years)	0.374 (0.151)	0.013	0.398 (0.183)	0.029
BMI (kg/m^2^)	−0.206 (0.169)	0.221		
Estradiol	−0.012 (0.022)	0.581	−0.026 (0.077)	0.734
TT	0.001 (0.002)	0.582	−0.020 (0.026)	0.453
FT	0.010 (0.057)	0.859	0.011 (0.012)	0.883
SHBG	0.743 (1.352)	0.583	0.099 (0.533)	0.853
SAH	1.138 (1.435)	0.427		
DM	−0.214 (1.421)	0.882		
CCI	−0.333 (1.353)	0.806		

BMI, Body Mass Index; TT, Total Testosterone; FT, Free Testosterone; SHBG, Sex Hormone-Binding Globulin; SAH, Systemic Arterial Hypertension; DM, Diabetes Mellitus; CCI, Cardiac Chronic Insufficiency; β, Estimated Coefficient; SE, Standard Error.

## Discussion

This cross-sectional observational study included 60 elderly men enrolled consecutively at the Geriatrics Service of the Hospital das Clínicas, University of São Paulo Medical School. Compared to most previous investigations, the sample comprised individuals of more advanced age and was evaluated comprehensively in a multidisciplinary setting. All participants were clinically stable outpatients, and cognitive and clinical assessments were conducted following standardized protocols by trained professionals, ensuring methodological consistency and minimizing information bias. This approach enhances the internal validity of the findings, despite the inherent limitations of the study design.

The relationship between serum testosterone levels and aging appears to be bidirectional, particularly when it comes to body composition. The aging process naturally leads to a decrease in lean muscle mass and an accumulation of body fat. These changes promote adipocyte‐aromatase‐mediated conversion of testosterone to estradiol, declining circulating testosterone levels.^20^ The increasing global prevalence of obesity further exacerbates this issue, as excess body fat contributes to this hormonal imbalance.^21^ Additionally, lower testosterone levels also contribute to muscle loss, since testosterone is crucial for stimulating protein synthesis and preventing muscle degradation.^22^^,^^23^

As anticipated, the authors observed a negative relationship between serum total testosterone levels and BMI. Another finding was a negative correlation between BMI and total MMSE score, which could represent an indirect influence of BMI on serum testosterone levels. Several potential mechanisms may mediate this relationship; adipose tissue is known to act as an endocrine organ, producing proinflammatory cytokines such as IL-6 and TNF-α, which may contribute to chronic low-grade inflammation and neuroinflammation.^23^^,^^24^ Moreover, increased visceral fat has been associated with structural brain changes and accelerated cerebral aging, particularly in regions involved in cognitive control and memory.^25^ Obesity is also a well-established risk factor for cerebrovascular damage, insulin resistance, and endothelial dysfunction, all of which may negatively impact cognitive performance.^24^ Furthermore, it is well established that excess adiposity in men is associated with reduced testosterone levels through suppression of the hypothalamic-pituitary-gonadal axis, potentially leading to functional hypogonadotropic hypogonadism.^23^ Therefore, we speculated that the association between higher BMI and lower cognitive scores could reflect a complex interaction of metabolic, vascular, and inflammatory processes that collectively contribute to neural compromise. Although the bivariate analyses showed associations between BMI and MMSE, this finding was not sustained in the adjusted model, highlighting the need to reassess in larger cohorts.

Interestingly, the authors identified a positive correlation between serum total testosterone levels and years of schooling, which has not evaluated in the literature. This could suggest that higher education levels influence lifestyle habits and/or access to healthy food, a hypothesis further supported by the negative correlation between schooling and BMI.^26^^,^^27^ In line with this, a recent study found that the association between educational attainment and BMI in older adults is partially mediated by lifestyle factors such as screen time and smoking, highlighting the role of behavioral choices in shaping metabolic health.^28^

Despite exploratory correlations, the multivariable analysis confirmed that serum testosterone levels, both total and free, were not independently associated with global cognitive performance as measured by the MMSE. Among the tested variables, only years of education remained a significant predictor of MMSE scores (β = 0.398; *p* = 0.029), underscoring the enduring cognitive reserve conferred by educational attainment.

Considering the data mentioned above, the authors cannot rule out the possibility that the lack of a statistically significant correlation between total MMSE scores and serum total testosterone levels may be due to the limited sample size or the sampling design employed. This interpretation is further supported by the multivariable regression model, in which testosterone was not independently associated with MMSE total scores after adjusting for age, education, BMI, and comorbidities, suggesting either a lack of effect or insufficient power to detect small associations. However, 11 out of 20 men (55 %) with total testosterone levels below 300 ng/dL exhibited impaired total MMSE scores. Additionally, total testosterone levels were positively associated with performance in the Spatial Orientation domain, a finding consistent with previous studies in this field.^11^^,^^29^ Despite these observations, a systematic review that analyzed nineteen studies published between 2000 and 2020 concluded that testosterone supplementation generally does not exert protective effects on cognition, as most clinical trials failed to demonstrate significant improvements in global cognitive function among elderly men with cognitive impairment and hypogonadism.^7^

Aging is also associated with a higher frequency of depression, and etiologic factors involved have been analyzed, including serum steroid levels. The relationship between the decline in serum testosterone levels and mood changes has been extensively studied with controversial findings. Some studies find an association between depressed mood and decreased testosterone levels, some reported that testosterone therapy improves depressive symptoms, while others have not observed these correlations.^7^^,^^30^, ^31^, ^32^

The authors identified a negative correlation among serum total testosterone, free testosterone levels and GDS scores; it is important to note that the cohort consisted of patients over 60-years-old, all of them with good clinical control of chronic diseases. Personal characteristics can influence GDS scores, as each one-unit increase in serum free testosterone levels was associated with a 0.12-point reduction in GDS scores. The authors hypothesized that different findings in the literature regarding the correlation between serum total testosterone levels and GDS scores could result from samples with different ages, including both young adults and older individuals, and from the use of different psychological tests, which complicates comparisons across studies.

While the present findings suggest an association between serum testosterone levels and depressive symptoms, particularly in the spatial orientation domain of cognition, the relationship between testosterone replacement and mental health outcomes remains controversial. For instance, the TRAVERSE trial,^12^ a large randomized controlled study involving 5204 hypogonadal men, reported only modest improvements in mood and energy following Testosterone-Replacement Therapy (TRT), with no significant benefit in cognitive function or sleep quality when compared to placebo. Moreover, in men with rigorously defined Low-Grade Persistent Depressive Disorder (LG-PDD), TRT did not lead to significant improvements in depressive symptoms or remission rates, possibly due to the limited sample size within this subgroup. Similarly, Resnick et al.,^31^ in the TTrials cognition study found no significant cognitive benefit of TRT in older men with age-associated memory impairment, despite measurable increases in serum testosterone. More recently, a 2025 meta-analysis reported moderate improvements in memory and executive function with TRT in aging men, while global cognition remained unchanged and publication bias was noted.^33^ A 2024 cross-sectional study by Tang et al. also described a non-linear “inverted U-shaped” association between testosterone levels and MMSE scores, particularly in men with vascular risk factors.^34^ These contrasting results underscore the complexity of the hormonal modulation of mood and cognition, and the need to consider individual variability, baseline cognitive status, and the presence of clinical depression when interpreting these associations.

The present study has limitations, including a small sample size and the presence of comorbidities among participants, which may have limited the statistical power to detect subtle associations and increased the risk of type II error. This is particularly relevant to the MMSE outcome, where the lack of association with testosterone levels in adjusted models may reflect insufficient power rather than a true null effect. The relatively wide confidence intervals and modest β coefficients observed in the regression further support the need for larger, well-controlled studies. Additionally, the cross-sectional design precludes causal inference and highlights the need for longitudinal studies to clarify the directionality of the associations observed. The authors also lacked structured data on medication use (e.g., therapy duration and daily doses), and although physical activity was assessed through IPAQ, it was not included in regression models, which may have limited the control of potential confounding effects.

Nonetheless, the study had important strengths: cognitive assessments were conducted by a single trained professional, and clinical evaluations were performed by a physician blinded to the participants' hormonal data. Moreover, the authors carried out a detailed analysis of MMSE subdomains, a rarely explored approach in this field, which may offer valuable insights for future prospective investigations.

In conclusion, this study observed that serum testosterone levels were positively associated with spatial orientation and negatively correlated with depressive symptoms. Interestingly, the key finding of this study was that the level of education showed a positive correlation with overall MMSE performance and a negative correlation with BMI, which may reflect an influence of lifestyle habits. These findings underscore the importance of adopting a comprehensive approach to the interplay between metabolic, hormonal, and cognitive factors in older adults, to enable early identification and intervention in modifiable domains, especially as the prevalence of cognitive impairment and dementia is expected to increase significantly in the coming decades.

## Funding

CCBC and JMLI were supported by grants from 10.13039/501100002322CAPES #1504183 and FAPESP #2023/16302-8, respectively, TASSB by grants from 10.13039/501100003593CNPq #308871/2022-7. This work was partially supported by grants from FAPESP #2019/26780-9 and #2024/00182-6.

## Data availability

The datasets generated and/or analyzed during the current study are available from the corresponding author upon reasonable request.

## CRediT authorship contribution statement

**Carolina C.B. Caetano:** Investigation, Data curation, Writing – original draft. **Sami Liberman:** Formal analysis, Methodology, Conceptualization. **Jéssica M.L. Ie:** Formal analysis, Writing – original draft, Visualization. **Valmari C.A. Toscano:** Investigation, Resources. **Wilson Jacob:** Project administration, Resources. **Tânia A.S.S. Bachega:** Conceptualization, Supervision, Validation, Writing – review & editing.

## Conflicts of interest

The authors declare no conflicts of interest.

## References

[1] Instituto Brasileiro de Geografia e Estatística (IBGE) (2023). Demographic and socio-economic characteristics of the population. Stat Annu Braz.

[2] Instituto Brasileiro de Geografia e Estatística (IBGE). Directorate of Surveys. Coordination of Population and Social Indicators (2018). https://www.ibge.gov.br/estatisticas/sociais/populacao/9109-projecao-da-populacao.html.

[3] Zhang Z., Kang D., Li H. (2020). Testosterone and cognitive impairment or dementia in middle-aged or aging males: causation and intervention, a systematic review and meta-analysis. J Geriatr Psychiatry Neurol.

[4] Maier A., Riedel-Heller S.G., Pabst A., Luppa M. (2021). Risk factors and protective factors of depression in older people 65+: a systematic review. PLoS One.

[5] Yeap B.B., Flicker L. (2022). Testosterone, cognitive decline and dementia in ageing men. Rev Endocr Metab Disord.

[6] Wang X., Lv Z., Wu Q., Liu H., Gu Y., Ye T. (2021). Lower plasma total testosterone levels were associated with steeper decline in brain glucose metabolism in non-demented older men. Front Aging Neurosci.

[7] Lisco G., Giagulli V.A., De Tullio A., De Pergola G., Guastamacchia E., Triggiani V. (2020). Age-related male hypogonadism and cognitive impairment in the elderly: focus on the effects of testosterone replacement therapy on cognition. Geriatr (Basel).

[8] Gregori G., Celli A., Barnouin Y., Paudyal A., Armamento-Villareal R., Napoli N. (2021). Cognitive response to testosterone replacement added to intensive lifestyle intervention in older men with obesity and hypogonadism: prespecified secondary analyses of a randomized clinical trial. Am J Clin Nutr.

[9] Tan S., Sohrabi H.R., Weinborn M., Tegg M., Bucks R.S., Taddei K. (2019). Effects of testosterone supplementation on separate cognitive domains in cognitively healthy older men: a meta-analysis of current randomized clinical trials. Am J Geriatr Psychiatry.

[10] McBride J.A., Carson C.C., Coward R.M (2016). Testosterone deficiency in the aging male. Ther Adv Urol.

[11] Hsu B., Cumming R.G., Waite L.M., Blyth F.M., Naganathan V., Le Couteur D.G. (2015). Longitudinal relationships between reproductive hormones and cognitive decline in older men: the Concord health and ageing in men project. J Clin Endocrinol Metab.

[12] Bhasin S., Seidman S., Travison T.G., Pencina K.M., Lincoff A.M., Nissen S.E. (2024). Depressive syndromes in men with hypogonadism in the TRAVERSE trial: response to testosterone-replacement therapy. J Clin Endocrinol Metab.

[13] Snyder P.J. (2022). Symptoms of late-onset hypogonadism in men. Endocrinol Metab Clin North Am.

[14] Psychiatric Association A. Manual diagnóstico e estatístico de transtornos mentais: DSM-5. 5ª ed. Artmed; 2014.

[15] Sebastião E., Gobbi S., Chodzko-Zajko W., Schwingel A., Papini C.B., Kokubun E. (2012). The international physical activity questionnaire-long form overestimates self-reported physical activity of Brazilian adults. Public Health.

[16] Folstein M.F., Folstein S.E., McHugh P.R. (1975). Mini-mental state”: a practical method for grading the cognitive state of patients for the clinician. J Psychiatr Res.

[17] de Melo D.M., Barbosa A.J.G. (2015). O uso do mini-exame do estado mental em pesquisas com idosos no Brasil: uma revisão sistemática. Ciênc Saúde Coletiva.

[18] Yesavage J.A., Brink T.L., Rose T.L., Lum O., Huang V., Adey M., Leirer V.O (1983). Development and validation of a geriatric depression screening scale: a preliminary report. J Psychiatr Res.

[19] Chachamovich E., Fleck M.P., Power M. (2010). Is geriatric depression scale-15 a suitable instrument for measuring depression in Brazil? Results of a Rasch analysis. Psychol Health Med.

[20] Jayasena C.N., Anderson R.A., Llahana S., Barth J.H., MacKenzie F., Wilkes S. (2022). Society for Endocrinology guidelines for testosterone replacement therapy in male hypogonadism. Clin Endocrinol (Oxf).

[21] Phelps N.H., Singleton R.K., Zhou B., Heap R.A., Mishra A., Bennett J.E. (2024). Worldwide trends in underweight and obesity from 1990 to 2022: a pooled analysis of 3663 population-representative studies with 222 million children, adolescents, and adults. Lancet.

[22] Parahiba S.M., Ribeiro É.C.T., Corrêa C., Bieger P., Perry I.S., Souza G.C. (2020). Effect of testosterone supplementation on sarcopenic components in middle-aged and elderly men: a systematic review and meta-analysis. Exp Gerontol.

[23] Pivonello R., Menafra D., Riccio E., Garifalos F., Mazzella M., De Angelis C. (2019). Metabolic disorders and male hypogonadotropic hypogonadism. Front Endocrinol (Lausanne).

[24] Morys F., Dadar M., Dagher A. (2021). Association between midlife obesity and its metabolic consequences, cerebrovascular disease, and cognitive decline. J Clin Endocrinol Metab.

[25] Moran C., Herson J., Than S., Collyer T., Beare R., Syed S. (2024). Interactions between age, sex and visceral adipose tissue on brain ageing. Diabetes Obes Metab.

[26] Centers for Disease Control and Prevention (2023). https://www.cdc.gov/obesity/php/data-research/adult-obesity-prevalence-maps.html.

[27] Ngandu T., Lehtisalo J., Solomon A., Levälahti E., Ahtiluoto S., Antikainen R. (2015). A 2-year multidomain intervention of diet, exercise, cognitive training, and vascular risk monitoring versus control to prevent cognitive decline in at-risk elderly people (FINGER): a randomised controlled trial. Lancet.

[28] Wang L., Ren J., Chen J., Gao R., Bai B., An H. (2022). Lifestyle choices mediate the association between educational attainment and BMI in older adults in China: a cross-sectional study. Front Public Health.

[29] Cai Z., Li H. (2020). An updated review: androgens and cognitive impairment in older men. Front Endocrinol (Lausanne).

[30] Wahjoepramono E.J., Asih P.R., Aniwiyanti V., Taddei K., Dhaliwal S.S., Fuller S.J. (2016). The effects of testosterone supplementation on cognitive functioning in older men. CNS Neurol Disord Drug Targets.

[31] Resnick S.M., Matsumoto A.M., Stephens-Shields A.J., Ellenberg S.S., Gill T.M., Shumaker S.A. (2017). Testosterone treatment and cognitive function in older men with low testosterone and age-associated memory impairment. JAMA.

[32] Handelsman D.J., Wittert G.A. (2024). Testosterone and depression symptoms in aging men. J Clin Endocrinol Metab.

[33] Wang Y., Lin H., Zhao W. (2025). Effects of testosterone therapy on cognitive function in aging men: a systematic review and meta-analysis. Biomed Rep.

[34] Tang Q., Zhao Y., Li M. (2024). Non-linear association between testosterone levels and cognitive performance in elderly men: a cross-sectional study. BMC Geriatr.

